# circSnx12 Is Involved in Ferroptosis During Heart Failure by Targeting miR-224-5p

**DOI:** 10.3389/fcvm.2021.656093

**Published:** 2021-04-21

**Authors:** Haoyuan Zheng, Lin Shi, Changci Tong, Yunen Liu, Mingxiao Hou

**Affiliations:** ^1^Laboratory of Rescue Center of Severe Wound and Trauma Chinese People's Liberation Army, Department of Cardiovascular Surgery, General Hospital of Northern Theater Command of China Medical University, Shenyang, China; ^2^Laboratory of Rescue Center of Severe Wound and Trauma Chinese People's Liberation Army, Emergency Medicine Department of General Hospital of Northern Theater Command, Shenyang, China; ^3^The Second Affiliated Hospital of Shenyang Medical College, The Veterans General Hospital of Liaoning Province, Shenyang, China; ^4^Shenyang Medical College, Shenyang, China

**Keywords:** heart failure, RNA, untranslated, computational biology, ferroptosis

## Abstract

Circular RNA (circRNA) is a subclass of non-coding RNAs that enables the circular transcripts resistant to the exonuclease digestion. Iron homeostasis is essential for the body to maintain normal physiological functions. At present, the relationship among circRNA, iron metabolism and heart failure remains largely unknown. This study aimed to explore the regulatory mechanism of circRNA and iron metabolism in heart failure. We obtained circRNA, miRNA and mRNA data from public databases and built a ceRNA network. The prediction results were verified in the myocardial tissues of pressure overload-induced heart failure mice through the use of histopathological staining methods, iron and malondialdehyde (MDA) measurement tests, quantitative real-time PCR (qRT-PCR), Western blot analysis and luciferase reporter assay. A total of 4 genes related to iron metabolism and oxidative stress were identified, and a ceRNA network involving 7 circRNAs, 7 miRNAs, and 4 mRNAs was constructed using bioinformatics tools. The results of qRT-PCR and Western blot analyses indicated that the expression level of FTH1 was similar with that predicted by bioinformatics analysis. Echocardiographic measurement showed that heart failure mice have lower fractional shortening and ejection fraction. Moreover, the myocardium of heart failure mice displayed obvious fibrosis as well as increased levels of iron and MDA compared to control mice. Besides, circSnx12 could act as an endogenous sponge to bind with miR-224-5p, and the 3'UTR region of FTH1 also had miRNA binding sites. A circRNA-miRNA-mRNA regulatory network was successfully constructed by identifying differentially expressed genes related to iron metabolism. This new approach reveals potential circRNA targets for the treatment of heart failure.

## Introduction

Heart failure (HF) has become a major healthcare burden worldwide, as it is one of the leading causes of morbidity and mortality globally. HF can be caused by myocardial infarction, myocarditis and hypertension. Prior research has made great progress in understanding the pathophysiological mechanisms of HF, but little is known about its gene-level alterations. Recent findings suggest that non-coding RNA can play a vital role in mediating the pathological process of HF. Thus, in-depth exploration of the molecular mechanisms underlying HF pathogenesis can provide new ideas for optimal HF therapy and gain new insights into HF biomarkers and drug development (1). At present, the common animal model of HF is transverse aortic constriction (TAC). By increasing the cardiac afterload, the myocardium of the mice becomes damaged and scarred, ultimately resulting in fibrotic. Hence, in this study, the expression profiling datasets of TAC-induced HF animal model were used for subsequent analysis. This model is the same as the modeling method in the datasets.

Circular RNA (circRNA) is a new type of non-coding RNA that has been discovered in plant viruses (2), and is generated by direct back-splicing of exons via covalent bonding. The lack of 5′ cap and 3′ poly A makes circRNA more resistant to exonuclease digestion, suggesting its better stability compared to linear RNA. Previous studies shown that circRNAs were involved in the pathogenesis of cardiovascular diseases, including vascular disease (3), atherosclerosis (4), cardiomyopathy (5, 6) and HF (7, 8). CircRNAs not only exhibit dynamic expression patterns in a series of physiological and pathological conditions, but also play significant roles in regulating gene expression and transcription (9–11). Recent decades have witnessed the great progress of miRNAs as one of the most significant molecular targets for studying the mechanisms of cardiovascular disease. David Port et al. (12) found that a number of miRNAs are differentially regulated in the failing heart, including miR-224. Annalisa Angelini et al. (13) obtained more than 2257 mature miRNA from endomyocardial biopsies. They found that miRNA profiling on formalin-fixed paraffin-embedded endomyocardial biopsies differentiates form different types of heart transplant rejection. Most endogenous circRNAs have conserved miRNA target sites that competitively bind miRNAs to mRNAs, thereby preventing miRNA-mediated post-translational inhibition and regulating the expression levels of target genes (14). Recent circRNA findings have provided new insights into the mechanisms of miRNA regulation in a broader cellular context. The circRNA CDR1 was the first circRNA identified to function as a miRNA sponge, which has 74 binding sites for miR-7 (15).

Iron is an essential element for various enzymatic reactions such as DNA replication and repair, cellular respiration and energy metabolism (16, 17). In the heart, systemic iron homeostasis is essential for maintaining normal cardiac function. However, excess iron in the cardiomyocytes can result in an uncontrolled condition called iron overload, which ultimately lead to adverse effects. At the cellular level, iron overload can increase the levels of reactive oxygen species (ROS), thus generating oxidative damage to large molecules such as DNA, membrane lipids and proteins (18). In addition, iron overload disrupts the dynamic balance between mitochondrial division and fusion in cells (19). Therefore, assessing the changes in these RNA molecules is critical to understanding the pathophysiological mechanisms of iron-induced HF. We constructed a circRNA-miRNA-mRNA network based on competitive endogenous RNA (ceRNA), which may be associated with iron overload during HF, by means of bioinformatics and experimental approaches.

## Materials and Methods

### Data Acquisition

Gene Expression Omnibus (GEO) is a public functional genomic database. We searched HF-related expression profiling datasets through GEO database and selected the miRNA array data of HF mice induced by pressure overload. MiRNA dataset (GSE99459) was downloaded from GEO, and the corresponding platform annotation information were also retrieved to convert the probe information into the corresponding gene name. Rudebusch et al. (20) obtained the protein expression profiles of HF through mass spectrometry (MS), and the raw date are available via ProteomeXchange with identifier PXD007171. Werfel et al. (21) obtained the circRNA expression data of HF through deep sequencing techniques and the raw date were published on PUBMED with identifier PMID27476877 (Table 1).

**Table 1:** Basic information of the 3 datasets.

**Date source**	**Datebase**	**ID**	**Sample type**	**Experiment type**	**Total number of quantified genes**
Circ RNA	PUBMED	PMID2747688	Mouse heart tissue	Deep sequencing	9,954
Mi RNA	GEO	GSE99459	Mouse heart tissue	Non-coding RNA profiling by array	928
Protein	ProteomeXchange	PXD007171	Mouse heart tissue	LC-MS/MS analysis	1,297

### Identification of Differential Expression Profiles of circRNAs, miRNAs, and mRNAs

Raw data were downloaded from GEO and PUBMED database. “Limma” package in R software was used to identify the differentially expressed miRNAs with thresholds of |logFC|>1 and *P* < 0.05. Both mRNA and circRNA data were extracted from the research results of Rudebusch et al. (20) and Werfel et al. (21), respectively, which had been uploaded to PUBMED.

### GO Functional Enrichment Analysis

UNIPROT (https://uniprot.org/) is publicly accessible database that consists of various biological resources and analysis tools. It also contains gene annotation and protein sequence data. To gain insights into the function of differentially expressed mRNAs in the network, UNIPROT database was employed for GO enrichment analysis. The significance level was set at *P* < 0.05. The R software packages ggplot2 and GOplot were used to visualize the results of the enrichment analysis.

### Prediction of circRNA-miRNA and miRNA-mRNA Binding Sites

Subsequently, TargetScan and RegRNA 2.0 database was utilized for assessing the regulatory relationships between miRNAs and mRNAs. Given that the miRNAs belonging to the same family may have similar seed sequences, only one miRNA ID was retained for each family group. To construct a ceRNA-based network, we screened miRNA-mRNAs with reverse expression relationships, as miRNAs often negatively regulate mRNA expression (22). Next, starBase database was used to predict the regulatory relationship between circRNAs and miRNAs. It is worth noting that the circRNA primarily functions as a miRNA sponge and its expression level may not affect miRNA expression. Finally, a circRNA-miRNA-mRNA regulatory network was established using Cytoscape 3.7.2 software based on the interactions of both miRNA-mRNA and circRNA-miRNA groups.

### Animal Models

The animal experiment protocol was approved by the ethics committee of the General Hospital of Shenyang Military Region. Six-week-old male C57/BL6J mice (weighting ~18–20 g) were supplied by the Shenyang Military Region General Hospital Experimental Animal Center. The mice were fed a standard diet and had unlimited access to drinking water. After 2 weeks of feeding, the mice were randomly assigned to TAC group (*n* = 15) and SHAM group (*n* = 16), and then anesthetized by intraperitoneal injection of 50 mg/kg pentobarbital sodium. To allow direct access to the transverse aorta, the mice a horizontal incision (5 mm in length) was made at the suprasternal notch. TAC operation was then performed by ligating the aorta between the right innominate and left carotid arteries using a 27G needle tied with 7-0 silk suture. The needle was promptly withdrawn, leaving the aortic constriction in place. The surgical procedure of mice in SHAM group was similar to that of mice in TAC group, except that the 7-0 silk suture was only crossed through the aortic arch without ligation.

### Echocardiographic Assessment

An echocardiogram was conducted on mice at 6 weeks post-TAC following anesthesia with 2.0% isoflurane and 1 L/min oxygen. Transthoracic echocardiographic images (two-dimensional and M-mode) were acquired using an ultrasound system (Vevo 2100, VisualSonics) equipped with a high-frequency (30 MHz) transducer. To evaluate left ventricular fractional shortening and ejection fraction, M-mode measurements were carried out in the short-axis view.

### Histopathological Analysis

Cardiac tissues were collected from 6 mice in each group, fixed in 10% neural-buffered formalin, embedded in paraffin, and then sectioned at 4-μm thickness. After hematoxylin and eosin (HE) staining, the tissue sections were examined using an optical microscope (OLYMPUS) at × 200 magnification. To evaluate myocardial fibrosis, the tissue sections were stained with a Masson's trichrome staining kit (BASO, BA-4079). Myocardium was stained red, nucleus were stained black and collagen fibers were stained blue. The collagen volume fraction was determined as the percentage of collagen (blue-stained area) to the total heart tissue area. Wheat germ agglutinin (WGA; GeneTex, GTX01502) staining was carried out to assess the size of the cardiomyocytes. All data were processed and analyzed by ImageJ software.

### Immunohistochemistry Analysis

Immunohistochemical staining was performed using an immunohistochemistry kit (MXB, KIT-9710). To inhibit endogenous peroxidase, the tissue sections were first exposed to 1% H_2_O_2_ and then subjected to heat-induced antigen retrieval at 97°C for 15 min. After incubation with GPX4, ACSL4, MYH6, and MYH7 primary antibodies for 1 h, the sections were rinsed with phosphate-buffered solution and incubated again with the corresponding secondary antibodies. Finally, the sections were stained with 3,3-diaminobenzidine (DAB; MXB, DAB-0031) and counterstained with hematoxylin, followed by microscopic examination. The cells with brownish-yellow particles in their nuclei and/or cytoplasm were classified as immunoreactive cells.

### Evaluation of Malondialdehyde (MDA) and Iron Concentrations

The levels of MDA and iron in mouse heart tissues were measured using the Lipid Peroxidation (MDA) Assay Kit (ab118970, Abcam) and Iron Assay Kit (MAK025, Sigma-Aldrich), respectively, according to the manufacturer's instructions.

### qRT-PCR Analysis

The myocardial tissues of 5 mice in each TAC group and SHAM group were used for qRT-PCR experiments. Total RNA was extracted from the myocardial tissues using Trizol reagent (Invitrogen), and cDNA was then synthesized using a First Strand cDNA synthesis kit (Geneseed). Quantitative RT-PCR analysis was conducted on a Real-Time PCR System (Applied Biosystems) using SYBR Green Master Mix (Geneseed), cDNA template and specific primers (Table 2). β-actin was used to normalize circRNA and mRNA expression, while U6 (also known as RNU6) was used to normalize miRNA expression. In the majority of miRNA expression studies, miRNAs or other small RNAs were used as reference genes (23). It is questionable as to whether mRNAs can be used reference genes in miRNA expression studies, because longer RNAs may have different isolation efficiencies compared to miRNAs, they may be degraded faster due to their length (24). So U6, as a snRNA, was used to mormalize miRNA expression. All reactions were performed in triplicate, and the relative expression levels of the target genes were determined by 2^−ΔΔCt^ method.

**Table 2:** Primer sequences of circRNA, miRNA, mRNA, and internal reference used for validation by qRT-PCR.

**Gene**	**Sequence(5′-3′)**
β-actin	F:GCTTCTAGGCGGACTGTTAC
	R:CCATGCCAATGTTGTCTCTT
FTH1	F:TAAAGAAACCAGACCGTGATGACT
	R:TGCAGTTCCAGTAGTGACTGATTC
ATP6V1A	F:GACGTCATCATCTATGTCGGCT
	R:ATGGACTCTACTTTCCCATCAACCT
circStip1	F:GCAACGAATGCTTCCAGAAA
	R:CTGCCTCTTCCTCTTCATCC
circMfsd6	F:TCTCTACTGGCATCTGGAAG
	R:CTTCAAAATTCTGAATGGTGATCTG
circTxnfc11	F:TCATCGGAGTTTTGGACCAATC
	R:CACGTCTTTTGCTCGACTCA
circTulp4	F:TGCAGAAGGCGCTACTATGA
	R:TCCTGGATGGACTCTTACAACTC
circSlc20a2	F:TGTACACAGGAGCGCCAGAA
	R:GCGCTGGACAGTGTTGAAGT
circSnx12	F:CTACGTCCCTGGGAAGACAA
	R:ACTATCTCGCTCCAGCTCAT
U6	F:CTCGCTTCGGCAGCACA
	R:AACGCTTCACGAATTTGCGT
miR-226-3p	RT:GTCGTATCCAGTGCAGGGTCCGAGGTATTCGCACTGGATACGACCGGAGAGCC
	F:ATGGTTCGTGGGGAGGGTTGGGTGGAG
	R:GTGCAGGGTCCGAGGT
miR-1306-5p	RT:GTCGTATCCAGTGCAGGGTCCGAGGTATTCGCACTGGATACGACCGGACGTTT
	F:ATGGTTCGTGGGCACCACCTCCCCTGC
	R:GTGCAGGGTCCGAGGT
miR-3082-5p	RT:GTCGTATCCAGTGCAGGGTCCGAGGTATTCGCACTGGATACGACCACACAGA
	F:ATGGTTCGTGGGGACAGAGTGTGTGTG
	R:GTGCAGGGTCCGAGGT
miR-671-5p	RT:GTCGTATCCAGTGCAGGGTCCGAGGTATTCGCACTGGATACGACCCTCCAGCC
	F:ATGGTTCGTGGGAGGAAGCCCTGGAGG
	R:GTGCAGGGTCCGAGGT
miR-224-5p	RT:GTCGTATCCAGTGCAGGGTCCGAGGTATTCGCACTGGATACGACCAACGGAA
	F:ATGGTTCGTGGGTAAGTCACTAGTGGT
	R:GTGCAGGGTCCGAGGT

### Western Blotting

The myocardial tissues were lysed with lysis buffer containing using protease and phosphatase inhibitors. Total protein content was assessed using BCA reagent. Equivalent amounts of protein extract were separated on SDS-PAGE, and subsequently transferred onto PVDF membranes. After blocking with 5% skim milk for 1 h, the membranes were incubated with primary antibodies such as ANP (Abcam, ab180649), GPX4 (Abcam, ab125066), NOX1 (Abcam, ab131088), ACSL4 (Abcam, ab155282), FTH1 (Abcam, ab183781), ATP6V1A (Abcam, ab199326), and GAPDH (Cell Signaling, 2118) overnight at 4°C. After washing, the membranes were incubated with HRP-conjugated secondary antibodies and visualized using an enhanced chemiluminescence substrate kit (Bio-Rad Laboratories).

### Dual-Luciferase Reporter Assay

To construct luciferase reporter plasmids, the fragments of FTH1 3'UTR and circSnx12 as well their mutant sequences were synthesized, and then introduced into a psiCHECK-2.0 vector. HL-1 cell line was cultured in 24-well plates and transfected with miR-224-5p mimic/luciferase reporter construct or negative control miRNA construct. After transfection for 48 h, the cells were quenched, and the luciferase reporter activity was determined using a dual-luciferase reporter assay system. Renilla luciferase was used as an internal normalization standard.

### Statistical Analysis

All results were presented as mean ± SD. Statistical differences between two groups were compared using the Student's *t*-test. A *p* < 0.05 was deemed statistically significant. All statistical tests were carried out by GraphPad Prism V. 6.0 and SPSS V. 26.0.

## Results

### Differential Expression Profiles of circRNAs, miRNAs, and mRNAs

**Figure 1:**
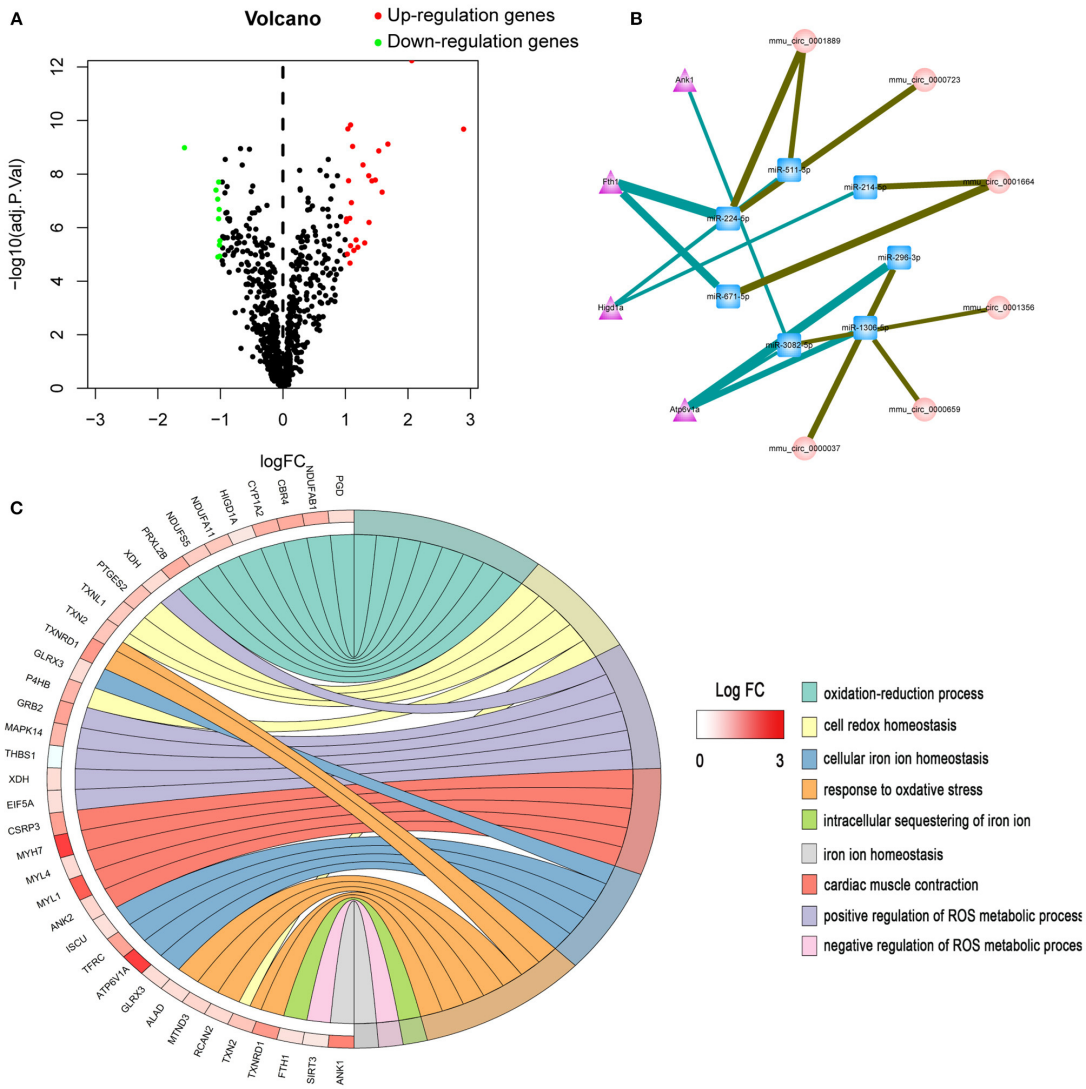
Bioinformatics analysis of differentially expressed genes. **(A)** Volcano plot of differentially expressed miRNAs. **(B)** The circRNA-miRNA-mRNA competing endogenous RNA network. The triangle nodes represent mRNAs in purple, circular nodes indicate circRNAs in pink, and square nodes represent miRNAs in blue. The thicker the edge, the higher the weight score of the interaction. **(C)** GO enrichment analysis of differentially expressed genes.

According to Julia Rudebusch's research (20), after TAC at day 28, the expression levels of 94 transcripts were altered in the left ventricle. Of them, 63 were up-regulated and 31 were down-regulated. In addition, Stanislas Werfel found that there were 96 up-regulated and 10 down-regulated circRNAs in the heart tissues of TAC mice as compared to SHAM mice. In this study, we downloaded miRNA expression datasets from GEO (GSE99459), and identified that 280 miRNAs were differentially expressed, including 183 up-regulated and 97 down-regulated miRNAs (Figure 1A, Supplementary Table 1).

### Screening of ceRNAs Associated With Iron Metabolism

Ferroptosis is an iron-dependent form of regulated cell death. Dysregulation of iron metabolism can lead to iron deficiencies and, eventually, human diseases. First, the mRNAs related to iron metabolism and oxidative stress were screened. GO enrichment analysis was performed on all differentially expressed mRNAs. In total, 37 mRNAs related to iron metabolism and oxidative stress were screened out (Figure 1C, Supplementary Table 2), among which 11 proteins showed significant changes within 28 days after TAC operation. Next, the most significantly differentially expressed genes (ATP6V1A, FTH1, HIGD1A, and ANK1) were chosen for target gene prediction. Based on the results of miRNA-mRNA interactions predicted by TargetScan and RegRNA 2.0, 7 differential expressed miRNAs and 4 differential expressed mRNAs were significantly interacted with each other. The results of circRNA-miRNA network prediction by starBase demonstrated that 7 circRNAs were interacting with the above miRNAs. Based on these findings, we constructed a circRNA-miRNA-mRNA network by using Cytoscape 3.8.0. A total of 7 circRNAs, 7 miRNAs and 4 mRNAs were included in the network (Figure 1B).

### TAC Mice Display Myocardial Injury and Fibrosis

**Figure 2:**
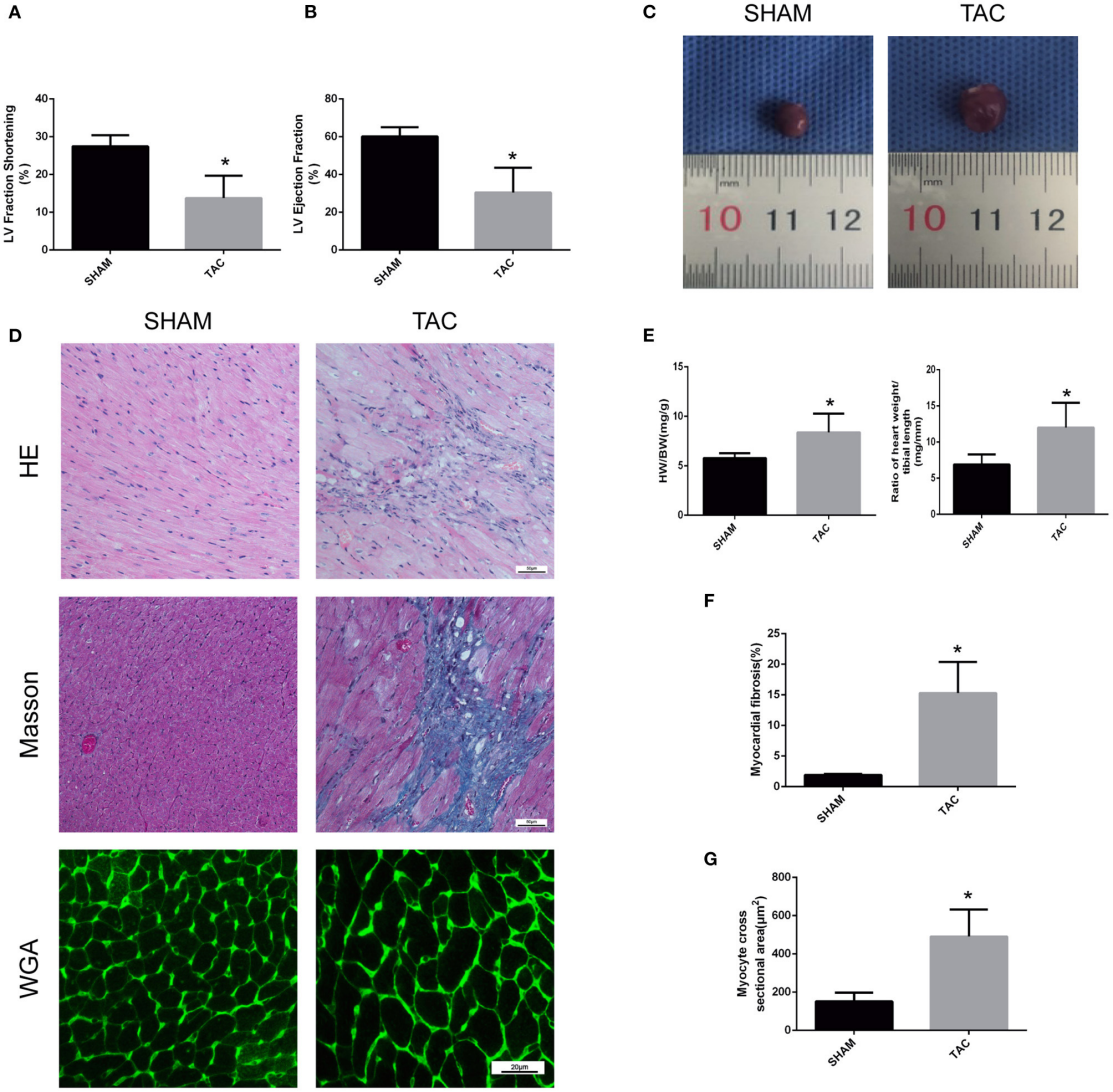
Functional and pathological analysis of heart tissues in TAC mice. **(A,B)** Left ventricular fraction shorting and ejection fraction. **(C)** Heart size. **(D)** Representative HE- and Masson-stained myocardial sections. WGA-stained myocardial sections were used to measure myocyte cross sectional area. **(E)** The heart weight/body weight and heart weight/tibia length ratios of TAC mice. **(F)** Quantitative data of myocardial fibrosis (%). **(G)** Quantitative data of myocyte cross sectional areas. *P < 0.05.

**Figure 3:**
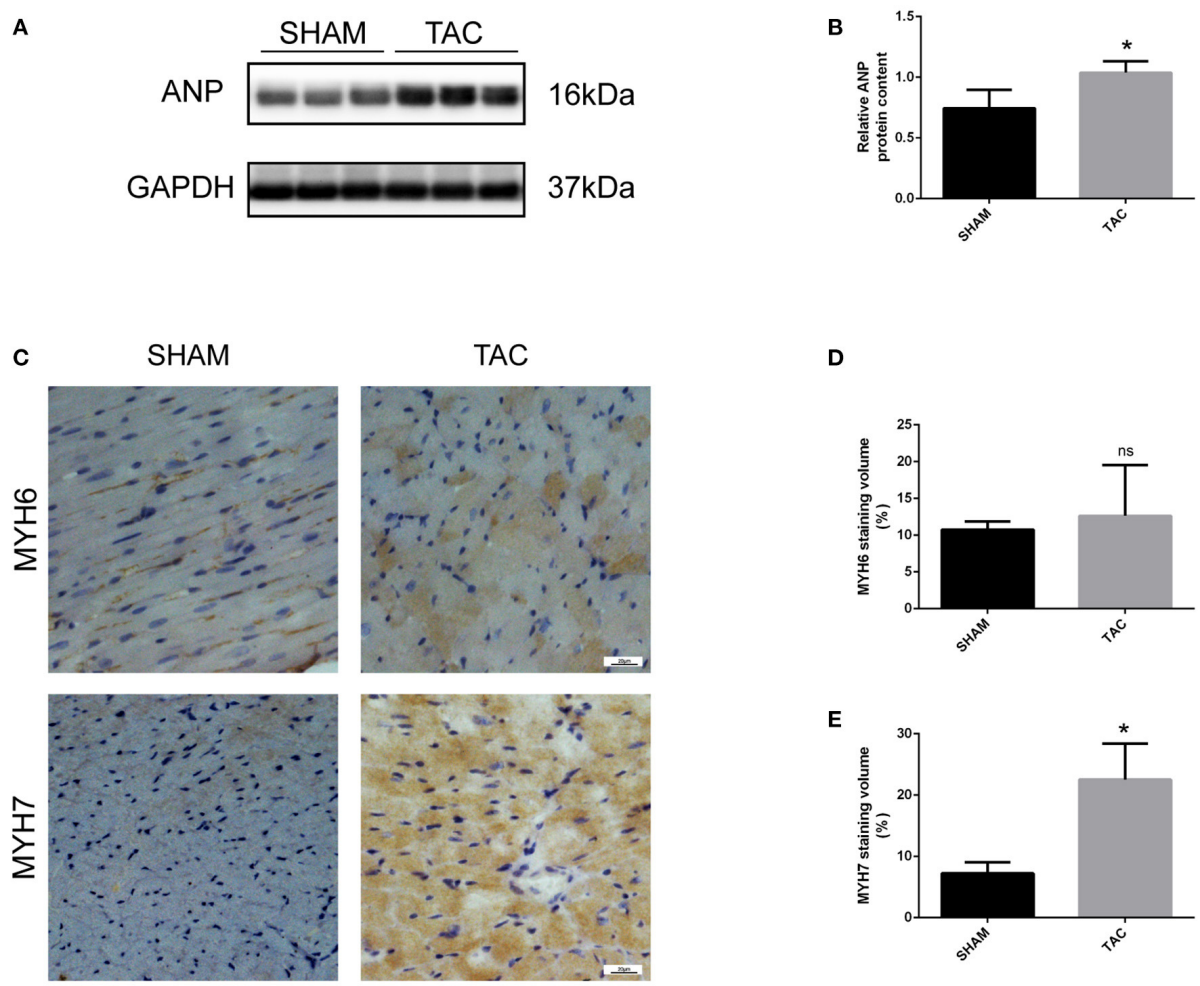
Western blotting and immunohistochemical analysis of TAC mice. **(A,B)** Representative Western blot image and quantitative data of ANP in myocardial tissues. **(C–E)** Representative immunohistochemical staining images and quantitative data of MYH6 and MYH7 in myocardial tissues. *P < 0.05; ns, non-significant.

In this experiment, we randomly divided C57/BL6J mice into two groups, namely TAC and SHAM. Echocardiographic measurement showed that TAC remarkably decreased both fractional shortening and ejection fraction in the left ventricle of HF mice (Figures 2A,B). The heart of the TAC group was larger than the heart of the SHAM group (Figure 2C). Meanwhile, chronic TAC significantly increased the left ventricular mass of HF mice (Figure 2E). Western blotting and immunohistochemical staining were carried out to identify the myocardial hypertrophy factors in mouse heart tissues. It was observed that the expression levels of ANP and MYH7 were markedly higher (*P* < 0.05) in TAC group than in SHAM group (Figures 3A–E). HE staining showed that cell necrosis and inflammatory cell infiltration were observed in the heart tissues of HF mice induced by pressure overload. Masson staining and WGA staining showed that the mice in TAC group had obvious myocardial fibrosis and myocyte hypertrophy (Figures 2D,F,G).

### Ferroptosis of Heart Tissue Is Induced by TAC

**Figure 4:**
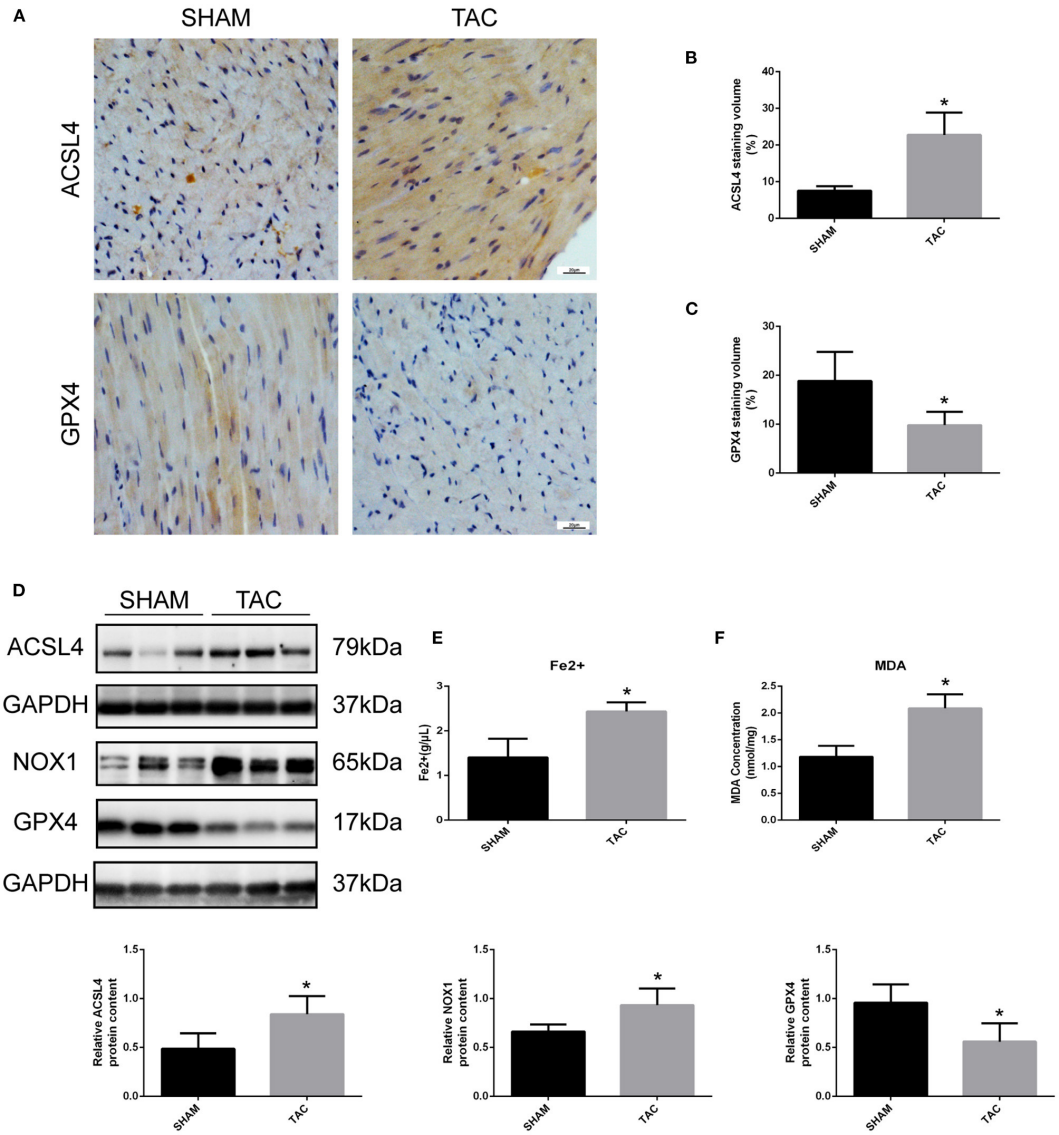
Detection of ferroptosis-associated genes in TAC mice. **(A–C)** Representative immunohistochemical staining images and quantitative data of ACSL4 and GPX4 in myocardial tissues. **(D)** Representative Western blot image and quantitative data of ACSL4, NOX1 and GPX4 in myocardial tissues. The concentrations of ferrous ion **(E)** and MDA **(F)** in TAC mouse heart tissues. *P < 0.05.

Western blot and immunohistochemistry analyses were performed to identify the regulatory factors of ferroptosis in mouse heart tissues and to determine the occurrence of ferroptotic-like death in TAC-induced HF mice. As shown in Figures 4A–D, the expression of GPX4 was decreased and the levels of ACSL4 and NOX1 were increased in TAC group when compared to SHAM group (*P* < 0.05). Furthermore, colorimetric assays were used to detect the content of ferrous ion and MDA in the mouse cardiomyocytes. Notably, the levels of ferrous and MDA were significantly higher in TAC group than in SHAM group (Figures 4E,F). This indicates that the myocardial tissues of mice in TAC surgery group are not only overloaded with iron, but also have increased lipid peroxidation.

### Analysis of the Candidate Genes Through qRT-PCR and Western Blot

**Figure 5:**
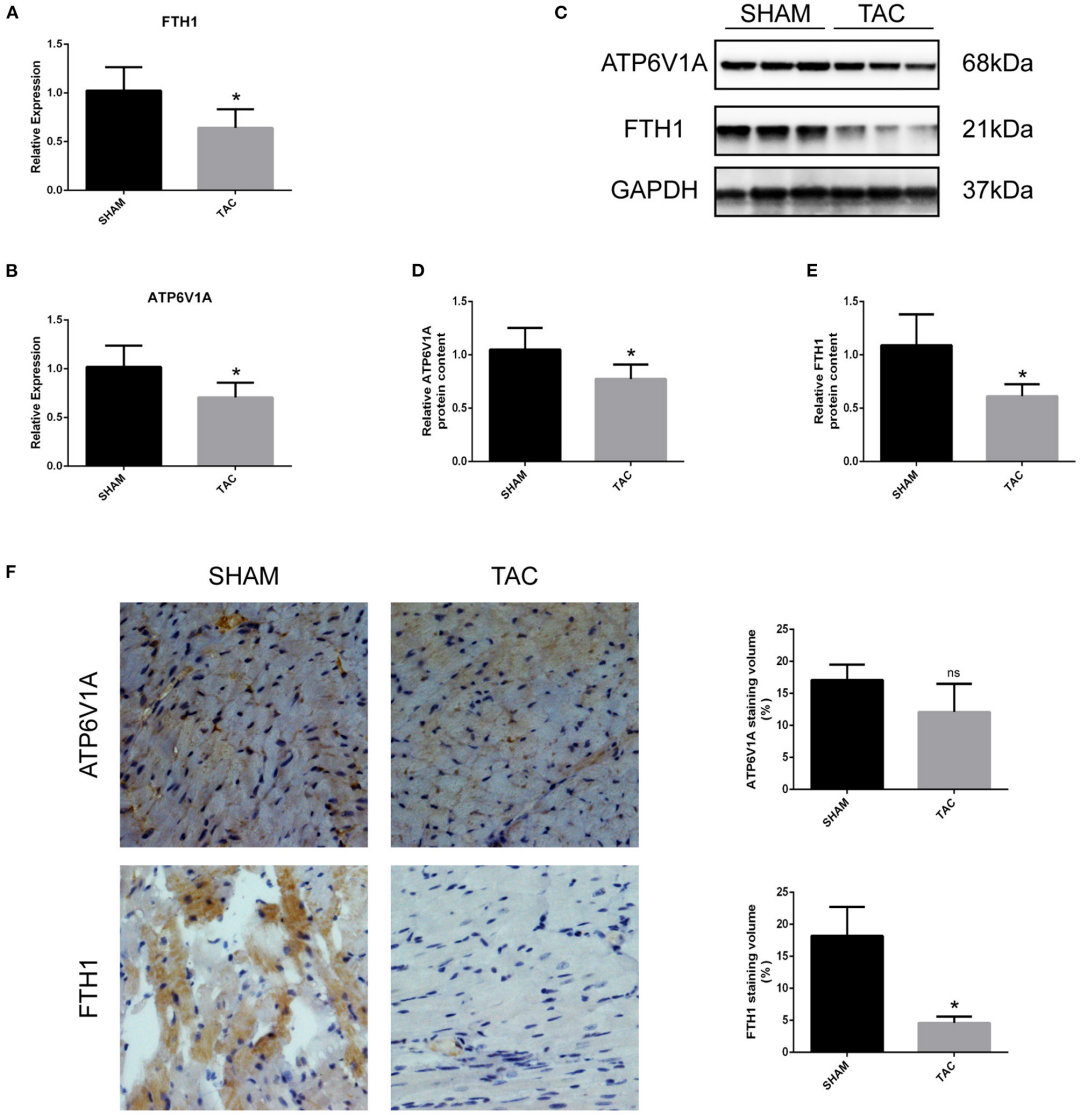
QRT-PCR and Western blot analyses of ATH6V1A and FTH1 in myocardial tissues. **(A,B)** Representative qRT-PCR results of FTH1 and ATP6V1A in myocardial tissues. **(C–F)** Representative Western blot images, immunohistochemistry, and quantitative data of ATP6V1A and FTH1 in myocardial tissues. *P < 0.05; ns, non-significant.

**Figure 6:**
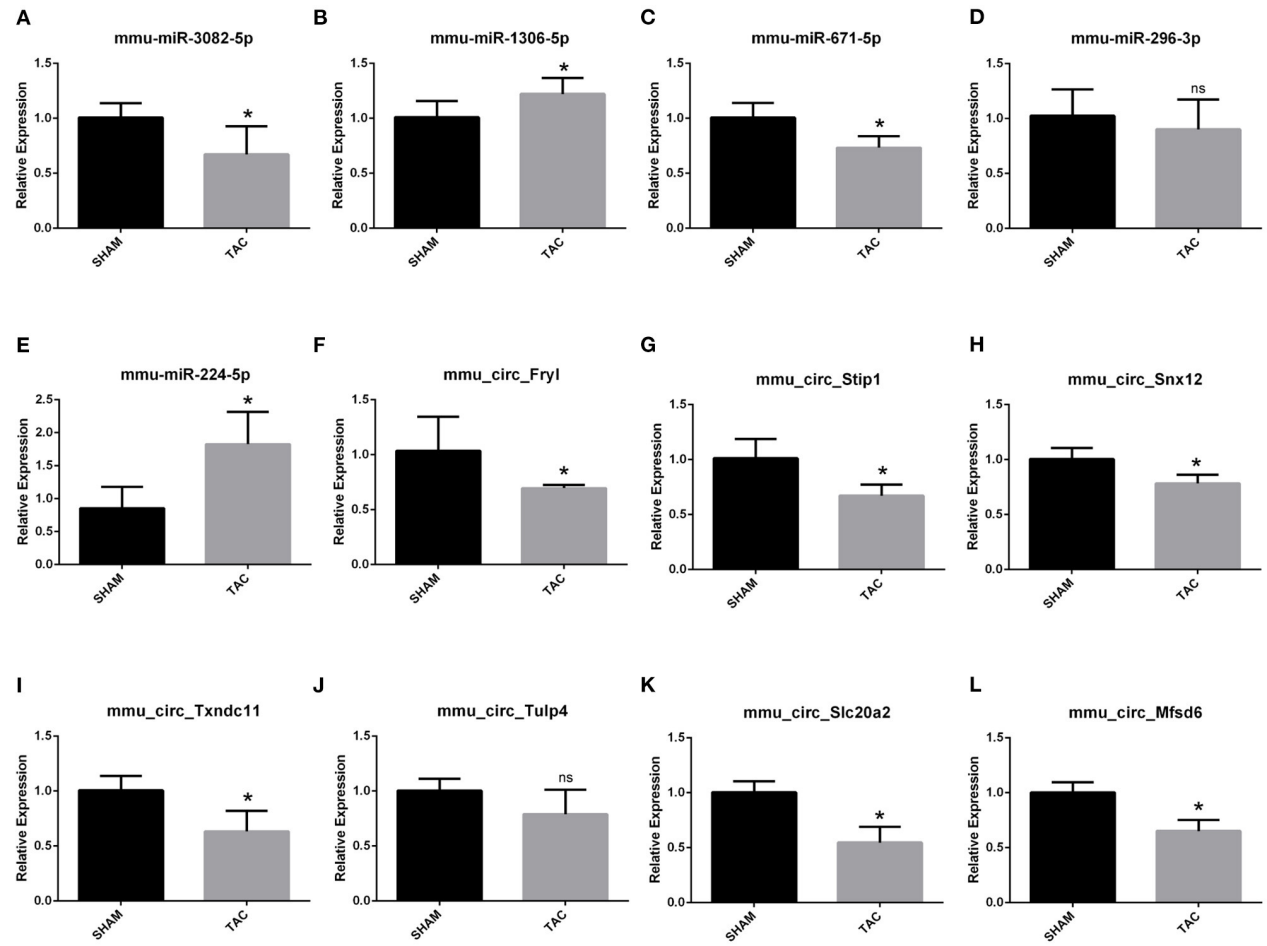
QRT-PCR validation of the differentially expressed circRNAs and miRNAs. The expression results of miRNAs **(A–E)** and circRNAs **(F–L)**. *P < 0.05; ns, non-significant.

Among the four candidate genes, we selected two ferroptosis-related genes (ATP6V1A and FTH1) for qRT-PCR and Western blot detection. In addition, 5 miRNAs and 7 circRNAs that may interact with ATP6V1A and FTH1 in the ceRNA network were selected for qRT-PCR verification. We found that FTH1 was down-regulated, as consistent with the target prediction results (Figure 5A). miR-3082-5p was down-regulate in the TAC group (Figure 6A), but miR-1306-5p and miR-224-5p was up-regulate (Figures 6B,E). There was no significant difference in the expression of miR-296-3p between the two groups (Figure 6D). In addition, circSLC20A2, circTULP4 and circSNX12, which may interact with FTH1, were also significantly down-regulated (Figures 6H,J,K). However, the expression pattern of miR-671-5p was contrary to the prediction results (Figure 6C). Moreover, the result of ATP6V1A expression was also contrary to the prediction results (Figure 5B), and the circRNAs (circSTIP1, circMFSD6, circTXNDC11 and circFRYL) that may interact with ATP6V1A were also significantly down-regulated (Figures 6F,G,I,L). Western blot analysis was performed to further confirm the protein levels of FTH1 and ATP6V1A. We found that the expression levels of FTH1 and ATP6V1A were lower in TAC group than in SHAM group (Figures 5C–F), which were consistent with the qRT-PCR data.

### miR-224-5p Directly Targets FTH1 and Interacts With circSnx12

**Figure 7:**
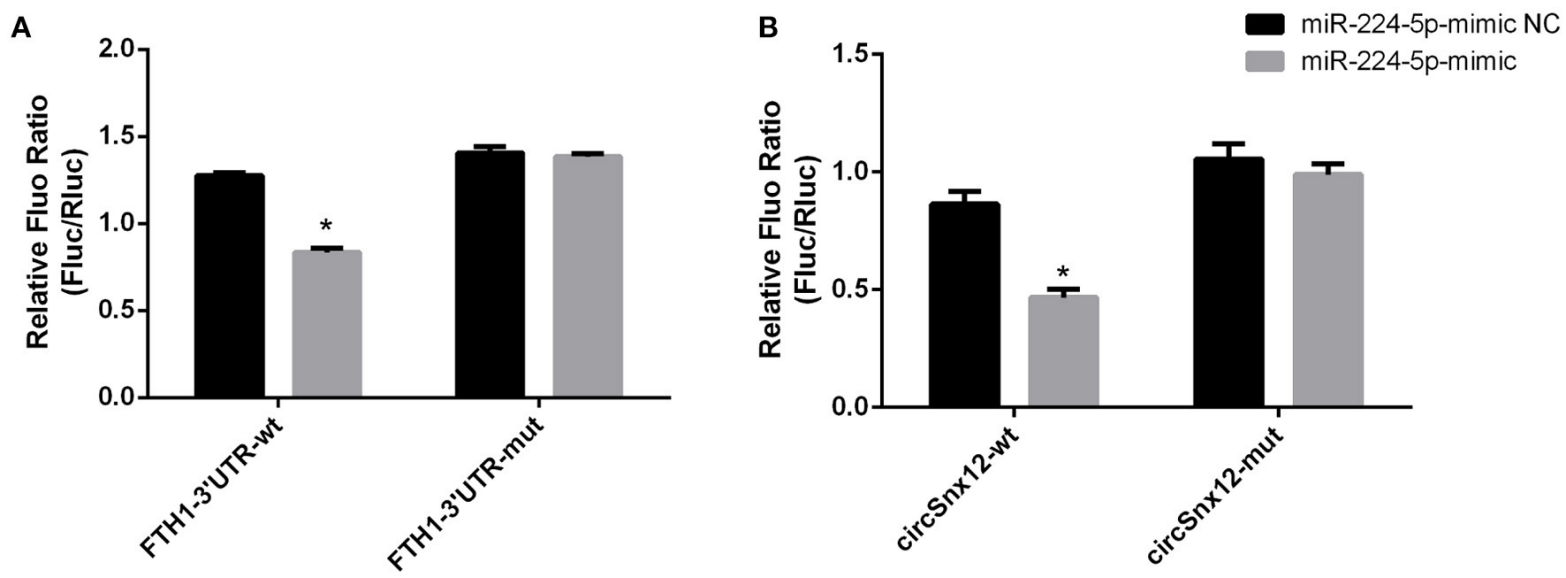
Dual-luciferase reporter activities. The interrelationships of miR-224-5p with FTH1 **(A)** and circSnx12 **(B)** were confirmed using dual-luciferase reporter assays. *P < 0.05.

Furthermore, we assessed the binding relationship between the ferroptosis-associated gene FTH1 and its target miR-224-5p. The results of luciferase reporter assay showed that miR-224-5p mimics could reduce the luciferase activity of FTH1 3′-UTR, but did not significantly affected that of FTH1 3′-UTR mutant (Figure 7A). These findings imply that FTH1 is a direct target of miR-224-5p. Additionally, we demonstrated the regulatory relationship between miR-224-5p and circSnx12. Sequence analysis revealed that there was a potential binding site for miR-224-5p in circSnx12 sequences. Consequently, wild-type and mutant circSnx12 were cloned into the psiCHECK2.0 vector and then co-transfected into HL-1 cells with miR-224-5p mimic or negative controls. The luciferase activity levels was remarkably lower in HL-1 cells co-transfected with miR-224-5p mimic and circSnx12 than in control group (Figure 7B). These findings suggest that miR-224-5p can directly interact with circSnx12.

## Discussion

The iron content in the mitochondria of cardiomyocytes is higher than that of other cells. Therefore, it is important to maintain the iron homeostasis of the mitochondria of cardiomyocytes (25). Iron overload can impair heart function by affecting cardiac mitochondrial dynamics and promoting cardiac mitochondrial deterioration (26). Chen et al. (27) indicated that cardiac cell death during HF can be reduced by inhibiting autophagy and ferroptosis. Through the use of HF mouse model induced by pressure overload, we found that the iron content of myocardial cells was noticeably increased compared to SHAM group. Moreover, the iron metabolism- and ferroptosis-related genes, such as FTH1, GPX4, NOX1, and ACSL4, were also differentially expressed. GPX4 is considered to be the primary enzyme that prevents ferroptosis (28). The decreased expression of GPX4 and increased expression of NOX1 and ACSL4 indicate that lipid peroxidation occurs in cardiomyocytes during HF, and the increase in MDA content also proves this phenomenon. Excessive iron accumulation in cardiomyocytes can generate ROS through Fenton reaction, thereby sensitizing cardiomyocytes to ferroptosis. In this study, we found that the expression levels of iron metabolism-associated genes were associated with HF, and these genes might interact with circRNAs and miRNAs. Moreover, we observed that ATP6V1A expression was down-regulated in the myocardium of TAC mice compared to SHAM mice, which appeared to be contrary to the bioinformatics prediction results. ATP6V1A is a catalytic subunit of the peripheral V1 complex of vacuolar ATPase that participates in systemic pH regulation in both lysosome and endosome (29). Lowering the pH of the endosome by ATP6V1A can release iron ions into the cytoplasm, thus disrupting systemic iron homeostasis in the body (30). Hence, downregulated ATP6V1A expression may be a sign of lysosomal acidification disorder. A recent study (31) showed that a decrease in V-ATPase-mediated lysosomal acidification could lead to the deposition of enlarged non-functional lysosomes and complete blockage of autophagic flux, which in turns causes damage to heart muscle cells. Another study (32) found that the abnormal expression of APT6V1A could dysregulate the function of V-ATPase in myocardial ischemia/reperfusion injury model, which in turn leads to myocardial injury and cell death. ROS could affect the function of lysosomes, thus leading to the accumulation of dysfunctional mitochondria and ultimately cell death (32). Nevertheless, the relationship between ATP6V1A and HF needs to be further verified by experiments. More importantly, we found that FTH1 was markedly down-regulated in TAC mice. FTH1 is an important component of ferritin. As a selective ferritin autophagic degradation process, ferritinophagy is also responsible for the regulation of systemic iron homeostasis and other iron-dependent pathophysiological processes (33, 34). Ferritin heavy chain (FHC) is involved in the first step of iron storage by catalyzing the ferroxidation of Fe^2+^; while ferritin light chain (FLC) enables the storage of Fe^3+^ by promoting the nucleation of ferrihydrite (35). Sun et al. (34) reported that the suppression of ferritinophagy by NCOA4 or Atg5/Atg7 siRNA could prevent pUL38-deficient human cytomegalovirus infection-induced cell apoptosis. Accumulating data have indicated that ferritinophagy is required for the induction of ferroptosis (36, 37). In this study, we found that the expression of FTH1 is repressed during HF, which in turn releases a large amount of ferrous ions into the cytoplasm. It has also been reported that excessive ferrous ions can increase the levels of ROS through the Fenton reaction, and radically attack cardiomyocyte mitochondria and cell membrane. Lipid peroxidation, as evidenced by the increased content of MDA, can contribute to cardiomyocyte dysfunction and ultimately cell death.

Growing evidence have suggested that circRNAs are important molecular regulators for HF pathogenesis. CircRNAs can act as miRNA sponges to counteract miRNA-mediated repression of mRNA, and thus interfering with its mRNA-targeting activity. However, it is unclear why linear RNA does not have a similar sponge function. CircRNAs can bind to several miRNAs or contain multiple binding sites for a single miRNA (38). In this study, we aimed to establish a putative regulatory network involving circRNAs, miRNAs and mRNAs in an animal model of HF induced by TAC. GO functional enrichment analysis revealed the potential roles of non-coding RNAs and coding RNAs in advanced HF. We first identified a protein associated with iron metabolism (FTH1), predicted that miR-224-5p might bind to FTH1 3'UTR by TargetScan and RegRNA 2.0 database, and further determined that miR-224-5p could be a potential binding target for circSnx12 through starBase database. The results of qRT-PCR verification indicated that circSnx12, miR-224-5p and FTH1 were correlated with each other, and double luciferase assay revealed that miR-224-5p was indeed the downstream target of circSnx12, and miR-224-5p could bind to the 3'UTR region of FTH1 and regulate its expression level. Therefore, it is speculated that low circSnx12 expression and high miR-224-5p expression can downregulate FTH1 expression, directly regulate iron overload in myocardial cells, and ultimately leads to cardiac cell death.

The changes in gene/protein expression can affect cell phenotypes. In this study, we determined the expression levels of proteins related to iron metabolism and oxidative stress, predicted the non-coding RNAs that interact with the target gene/proteins, constructed a ceRNA-based network, and performed experimental verification. A significant interrelationship among circRNA, miRNA and mRNA was identified in HF mice. However, the main limitation of our study was that we did not detect the expression level of circSnx12 that may affect miR-224-5p expression. In the near future, we will verify the effects of circSnx12 and miR-224-5p overexpression or knockdown on iron overload-induced mitochondrial abnormalities and myocardial cell death. In addition, we will prove the interaction between CircRNA and AGO2 by RNA pull-down and RNA IP to support that the revealed circRNAs can act as sponges of miRNAs.

## Conclusions

In summary, we successfully constructed a circRNA-miRNA-mRNA regulatory network through identification of iron metabolism-associated genes and further conducted experimental verification using TAC mouse model. This new approach reveals potential circRNA targets for the treatment of HF.

## Data Availability Statement

Publicly available datasets were analyzed in this study. This data can be found at: PUBMED(PMID2747688); GEO(GSE99459); ProteomeXchange(PXD007171).

## Ethics Statement

The animal study was reviewed and approved by Ethics Committee of the General Hospital of Northern Theater Command.

## Author Contributions

HZ collected the tissue sample, analyzed the data, and wrote the manuscript. LS and CT helped with histopathological staining. YL made the revision of the manuscript. MH made the revision of the manuscript and designed the research. All authors contributed to the article and approved the submitted version.

## Conflict of Interest

The authors declare that the research was conducted in the absence of any commercial or financial relationships that could be construed as a potential conflict of interest.
